# Infant Trauma Alters Social Buffering of Threat Learning: Emerging Role of Prefrontal Cortex in Preadolescence

**DOI:** 10.3389/fnbeh.2019.00132

**Published:** 2019-06-21

**Authors:** Patrese A. Robinson-Drummer, Maya Opendak, Anna Blomkvist, Stephanie Chan, Stephen Tan, Cecilia Delmer, Kira Wood, Aliza Sloan, Lily Jacobs, Eliana Fine, Divija Chopra, Chaim Sandler, Giselle Kamenetzky, Regina M. Sullivan

**Affiliations:** ^1^Emotional Brain Institute, Nathan Kline Institute for Psychiatric Research, Orangeburg, NY, United States; ^2^Department of Child and Adolescent Psychiatry, Child Study Center at NYU Langone Medical Center, NYU School of Medicine, New York, NY, United States; ^3^Department of Psychology, Stockholm University, Stockholm, Sweden; ^4^Department of Neural Science, New York University, New York, NY, United States; ^5^Department of Psychology, Florida Atlantic University, Boca Raton, FL, United States; ^6^Department of Biology, Yeshiva University, New York, NY, United States; ^7^Instituto de Investigaciones Médicas A Lanari, IDIM-CONICET, Universidad de Buenos Aires, Combatientes de Malvinas 3150 (CP 1427), Buenos Aires, Argentina

**Keywords:** early-life trauma, social buffering, social support, threat, fear, prefrontal cortex, infralimbic, prelimbic

## Abstract

Within the infant-caregiver attachment system, the primary caregiver holds potent reward value to the infant, exhibited by infants’ strong preference for approach responses and proximity-seeking towards the mother. A less well-understood feature of the attachment figure is the caregiver’s ability to reduce fear *via* social buffering, commonly associated with the notion of a “safe haven” in the developmental literature. Evidence suggests this infant system overlaps with the neural network supporting social buffering (attenuation) of fear in the adults of many species, a network known to involve the prefrontal cortex (PFC). Here, using odor-shock conditioning in young developing rats, we assessed when the infant system transitions to the adult-like PFC-dependent social buffering of threat system. Rat pups were odor-shock conditioned (0.55 mA–0.6 mA) at either postnatal day (PN18; dependent on mother) or 28 (newly independent, weaned at PN23). Within each age group, the mother was present or absent during conditioning, with PFC assessment following acquisition using ^14^C 2-DG autoradiography and cue testing the following day. Since the human literature suggests poor attachment attenuates the mother’s ability to socially buffer the infants, half of the pups at each age were reared with an abusive mother from PN8–12. The results showed that for typical control rearing, the mother attenuated fear in both PN18 and PN28 pups, although the PFC [infralimbic (IL) and ventral prelimbic (vPL) cortices] was only engaged at PN28. Abuse rearing completely disrupted social buffering of pups by the mother at PN18. The results from PN28 pups showed that while the mother modulated learning in both control and abuse-reared pups, the behavioral and PFC effects were attenuated after maltreatment. Our data suggest that pups transition to the adult-like PFC social support circuit after independence from the mother (PN28), and this circuit remains functional after early-life trauma, although its effectiveness appears reduced. This is in sharp contrast to the effects of early life trauma during infancy, where social buffering of the infant is more robustly impacted. We suggest that the infant social buffering circuit is disengaged by early-life trauma, while the adolescent PFC-dependent social buffering circuit may use a safety signal with unreliable safety value.

## Introduction

For infants, the mother and other significant caregivers serve as potent reward stimuli and induce robust proximity-seeking in the infant, regardless of the quality of care received. This infant attachment to the caregiver is learned during a sensitive period and rodent work suggests there is a unique neural network that robustly supports learning proximity-seeking (Moriceau et al., 2010; Raineki et al., 2010; Bisaz and Sullivan, 2012; Perry et al., 2016; Opendak et al., 2017). This open attachment system permits the infant to attach to multiple caregivers, including non-biological caregivers, within the context of diverse rearing conditions. Strikingly, this proximity-seeking characteristic of the attachment system is maintained even when the caregiver is the source of the threat, as occurs in maltreatment in a wide variety of species, including humans (Bowlby, 1982; Tottenham and Sheridan, 2009; Sanchez et al., 2015; Drury et al., 2016; Howell et al., 2017; Zajac et al., 2019).

A less well-known feature of the attachment figure is his or her ability to suppress or block fear/threat responding during early life, also referred to as social buffering (Hostinar et al., 2014; Gunnar et al., 2015; Hostinar and Gunnar, 2015; Callaghan et al., 2019). This fear reduction system was first characterized within Bowlby’s Attachment Theory (Bowlby, 1969, 1978) and is critical for the infant to approach the caregiver (safe base) for protection when threatened, rather than showing adult-like threat response behaviors (e.g., freezing, attacking or hiding; Coss, 2016). This phenomenon of social buffering of threat by the parent was first demonstrated in infant rats when the presence of the mother reduced the young infants’ responses to shock and blocked stress hormone release. This system is strongly phylogenetically represented and has been shown in rodents (Stanton and Levine, 1985; Levine et al., 1988; Suchecki et al., 1993; Hennessy et al., 2006, 2009, 2015; Gunnar et al., 2015; Sullivan and Perry, 2015; Al Aïn et al., 2017; Opendak et al., 2019), nonhuman primates and children (Coe et al., 1978; Wiener et al., 1987; Nachmias et al., 1996; Hennessy et al., 2009; Tottenham et al., 2012, accepted; Gee et al., 2013a; Sanchez et al., 2015; Howell et al., 2017). This social buffering supports the role of the attachment figure as a regulator of the immature infant (Bowlby, 1982; Hofer, 1994; Sroufe, 2005; Blair and Raver, 2015; Chambers, 2017; Feldman, 2017; Perry et al., 2017).

We have some understanding of the neural network supporting infant social buffering. This system involves caregiver suppression of the paraventricular nucleus (PVN) of the hypothalamus to block engagement of the stress axis (Shionoya et al., 2007) and attenuation of the amygdala and ventral tegmental response to threat (Hennessy et al., 2006, 2009; Moriceau and Sullivan, 2006; Moriceau et al., 2006, 2009; Opendak et al., 2019). This network analysis has, in part, been replicated in children (Gee et al., 2014; Tottenham et al., accepted), and nonhuman primates (Gunnar et al., 2015; Sanchez et al., 2015; Howell et al., 2017). Importantly, the literature across these species suggests that social buffering by maternal presence is disrupted in mother-infant dyads with poor quality attachment (Nachmias et al., 1996; Gunnar and Quevedo, 2007; Hostinar et al., 2014; Gunnar et al., 2015; Sanchez et al., 2015; Gunnar and Sullivan, 2017; Opendak et al., 2019). Yet, the neurobiology of this compromised social buffering system has received little attention.

Social buffering wanes with maturation, although this effect can still be seen in adults of many species. While there appears to be some overlap in the neural mechanisms across development, the late-developing prefrontal cortex (PFC) appears critical in adult social buffering (Hennessy et al., 2006, 2015, 2018; Kiyokawa et al., 2007, 2012; Taylor et al., 2008; Upton and Sullivan, 2010; Inagaki and Eisenberger, 2012; Tottenham et al., 2012; Hostinar et al., 2015; Hornstein et al., 2016; Harrison et al., 2017; Hornstein and Eisenberger, 2017). Here, we focus on the PFC and its evolving role in social buffering of the threat response, targeting a developmental transition from dependence on the mother (postnatal day [PN] 18) to independence in preadolescent rats (PN28) weaned from the mother. To further probe the dynamics of this developing circuit, we perturbed the system by exposing half of the animals to maternal maltreatment in early infancy. Overall, our results suggest that the neurobehavioral substrates of maternal social buffering and its perturbation are distinct during sensitive periods in development.

## Materials and Methods

### Subjects

A total of 322 Long Evans rats (178 PN18 ±1 day, 144 PN28 ±1 day), with approximately equal males and females, were bred and reared in our animal facility with *ad libitum* food and water. Animals were reared with an abusive mother or control mother from PN8-PN12–an age range documented to induce neurobehavioral deficits. Animals were tested at PN18 while still living with the mother or PN28 when pups live independently of the mother (all animal only tested once). Animals were always housed in an enclosure with solid floors, with both breeding and rearing occurring in a private animal room within the lab. Two weeks before giving birth, pregnant females were moved from large breeding cages to standard cages for birth and pup rearing (34 long × 29 wide × 17 high cm). General health and births were checked twice daily with the day of birth designated PN0. Litters were culled to 12 pups (approximately equal males and females) at PN1. Cages were cleaned twice a week except for the nest, which was saved and placed back with the mother and pups. All procedures were approved by the Institutional Animal Care and Use Committee in accordance with guidelines from the National Institutes of Health.

### Scarcity-Adversity Model of Low Bedding (LB; PN8–12)

**Figure 1:**
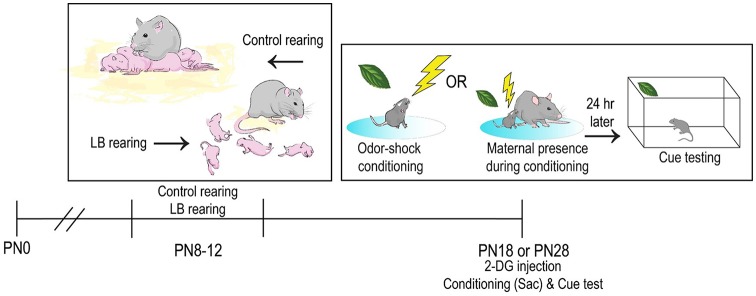
Schematic of methodology and experimental timeline. In infancy, pups received either Scarcity-Adversity Model of Low Bedding (LB) rearing or control rearing from the mother (ages PN8–12). LB rearing involved providing the mother with insufficient bedding for nest building, which produces maltreatment of pups but growth indistinguishable from controls. Pups are odor-shock conditioned in the mother’s presence or absence at one of two ages, with the goal of better understanding the neural mechanisms involved in social suppression of threat. A portion of the pups had the brain removed immediately after conditioning, while the other half were tested the next day (Cue test involving odor only presentations). The younger subjects were PN18, an age when pups are still with the mother but only for about 5 days before weaning. The other age tested was PN28, when pups have been independent for about 5 days.

Early-life trauma was modeled in rats using a well-established Scarcity-Adversity Model previously utilized by our lab and others (Sullivan et al., 2000; Raineki et al., 2010; Opendak and Sullivan, 2016; Opendak et al., 2017; Walker et al., 2017; Yan et al., 2017). As illustrated in Figure 1, the low bedding (LB) rearing takes place from PN8–12 and included the following manipulations: nest hutch removal, bedding material reduced from 4,000 mL to 100 mL and solid floor cage cleaned daily with bedding replaced to reduce odor and maintain a clean cage environment. As illustrated in Table 1, this procedure increases instances of maternal maltreatment of the pups (e.g., reduced time with pups, rough handling pups) and results in neurobehavioral dysfunction, including depressive-like behavior, disrupted social behavior and dysregulation of fear expression in pups, although major neurobehavioral effects show significant emergence at weaning age (Perry and Sullivan, 2014; Al Aïn et al., 2017; Opendak et al., 2017). Age-matched control litters were reared concurrently but with abundant bedding and nest-building materials. Pups were videotaped three times a week and data analyzed using Ethovision (Noldus Information Technologies Inc., Leesburg, VA, USA). Maternal behavior and infant-mother interactions were hand-scored using BORIS (Life Sciences and Systems Biology) behavioral coding software to validate abusive and non-abusive care.

**Table 1:** The Scarcity-Adversity Model of Low Bedding (LB) is a validated procedure of inducing abuse by providing the mother with insufficient nest building material.

	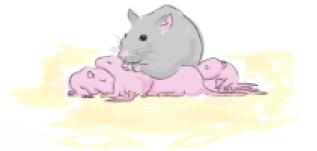	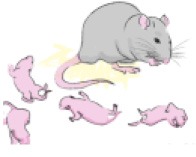
Maternal Behavior	Control % observations ± SEM	LB % observations ± SEM
*Nursing*	62.5 ± 23.8	76.7 ± 7.9
*In nest*	87.5 ± 10.2	91.1 ± 4.8
*Step on pups*	8.3 ± 8.3	50.0 ± 23.6
*Drag pups*	0 ± 0	27.8 ± 11.8
*Pups vocalize*	16.7 ± 9.6	64.4 ± 6.5

*Convergent with previous studies (Roth and Sullivan, 2005; Walker et al., 2017; Santiago et al., 2018) we observed increased rough handling of pups and increased pup vocalizations during a greater percentage of observations in the LB groups*.

### Odor-Shock Conditioning (Dependent on Mother PN18 or Independent PN28)

Conditioning took place in standard mouse fear conditioning (Coulbourn Instruments) apparatus within a sound attenuation chamber (Med Associates) with Coulbourn FreezeFrame software controlling stimuli delivery and video recording. Animals received a 20 min habituation session in the conditioning chambers a day prior to conditioning. On conditioning day, animals were given a 10 min adaptation period to the conditioning chamber before the start of conditioning. The conditioned stimulus (CS) was a 30 s peppermint odor (McCormick Pure Peppermint; 2 L/min; 1:10 peppermint vapor to air) controlled with a solenoid valve that minimized pressure changes by diverting airflow from the clean air to the peppermint air stream. To ventilate the chamber and ensure removal of odor CS, a standard attenuating chamber fan provided a constant stream of deodorized air flow through the chambers (2 L/min). The unconditioned stimulus (US) was a 1 s 0.6 mA foot shock delivered through a grid floor. The Paired experimental animals received a total of seven CS-US presentations administered at a 4 min inter-trial interval (ITI) and co-terminated with the 1 s footshock during the last second of the odor. Unpaired (behavioral control) animals received the same number of odor and shock presentations, however, the stimuli were separated by a 2 min inter-stimulus interval (ISI). Animals in the Odor-only condition also received the seven odor presentations but no shocks. Half of the experimental animals were conditioned in the presence of a urethane-anesthetized dam placed directly adjacent to the conditioning chamber where her odor was perceptible but she was not visible. Following conditioning, animals were either sacrificed and brains assessed for regional activity or retained for behavioral cue testing the next day to assess learning. PN18 and PN28 animals were only used at one age. These procedures were done according to published laboratory protocols (Boulanger Bertolus et al., 2014; Debiec and Sullivan, 2014; Tallot et al., 2016).

### Neural Assessment

Animals used for neural assessment were injected with ^14^C-labeled 2-deoxyglucose (2-DG; 20 μCi/100 g, i.p.) just prior to being placed in the conditioning chamber and brains removed after conditioning (45 min after injection). Brains were stored in a −80°C freezer before being sectioned in a cryostat (20 μm) at −20°C. Through the region of interest (ROI), every third slice was collected onto a coverslip and slices along with ^14^C standards (10 × 0.02 mCi, American Radiolabeled Chemicals Inc., St. Louis, MO, USA) were exposed to X-ray film (Kodak) for 5 days. The autoradiograph was then digitally scanned and prepared for analysis. All procedures occurred according to published lab protocols (Perry et al., 2016; Opendak et al., 2019).

### PFC Analysis

Autoradiographs were analyzed using ImageJ software (National Institutes of Health) for quantitative optical densitometry with an increase in autoradiographic density indicating increased 2-DG metabolism. Using Paxinos and Watson (2013) as a guide, two medial prefrontal regions were identified and analyzed for regional activity: Prelimbic (PL) and Infralimbic (IL), each of which was subdivided into additional subregions. At least three sections from the rostro-caudal extent were analyzed for each brain area.

Regional engagement levels were expressed as 2-DG uptake relative to that observed in white matter tracts (e.g., the anterior commissure or forceps minor) to control for differences in exposure levels or section thickness (Sullivan et al., 2000). Autoradiographic density was measured in both hemispheres of the brain for each region of interest and then averaged across both hemispheres, as no statistical difference was found between hemispheres.

### Cue Test

Twenty-four hours following conditioning, learning was assessed using a cue test in a new context: novel room, placed in a 5,000 mL glass beaker inside a sound attenuating chamber (Coulbourn) with the fan placed outside the attenuating box. Context was further changed by cleaning the attenuating chamber with Windex (SC Johnson) 5 min before animals were placed within the beaker. For cue testing, animals were placed in the beaker and given a 5 min acclimation period prior to the first odor onset. Five 30 s presentations of the peppermint odor were presented using a 4-min ITI, as described for conditioning. Learning was measured by total time (in seconds) freezing during the odor with freezing defined as the cessation of all body movements with the exception of that minimally required for breathing. Freezing was scored automatically by FreezeFrame, although all freezing was checked by a blind scorer to determine freezing vs. inactivity. All animals were videotaped using two cameras, a side view and a top view to ensure accurate behavioral scoring.

### Statistical Analysis

All behavior data were separated by age and rearing condition and analyzed using a two-way analysis of variance (ANOVA) with repeated measures [maternal presence (alone vs. with mom) × cue presentation (cue #1–5)] for training day data and two-way ANOVA [learning condition (paired, unpaired, odor only) × maternal presence (alone vs. with mom)] for cue test data, followed by Bonferroni-corrected pairwise tests. Planned comparisons were used when justified by a priori hypotheses (see Results section below). No sex effects or interactions were found in freezing behavior at either PN18 or PN28 and therefore data were collapsed across sex for analysis of maternal presence effects on behavior and 2-DG uptake. 2-DG uptake data were analyzed separately for each age using two-way ANOVA (rearing × maternal presence), followed by Bonferroni-corrected pairwise tests. All differences were considered significant when *p* < 0.05. All data analysis was performed by an experimenter blind to the experimental conditions.

## Results

### Mother-Infant Response to Scarcity-Adversity Model

Offline, blinded observations of videos of mother-infant interactions during control and LB Adversity-Rearing (PN8–12) indicated that the LB pups received more rough handling by the mother than controls (see Table 1 for further details).

### Odor-Shock Conditioning Acquisition Curves

**Figure 2:**
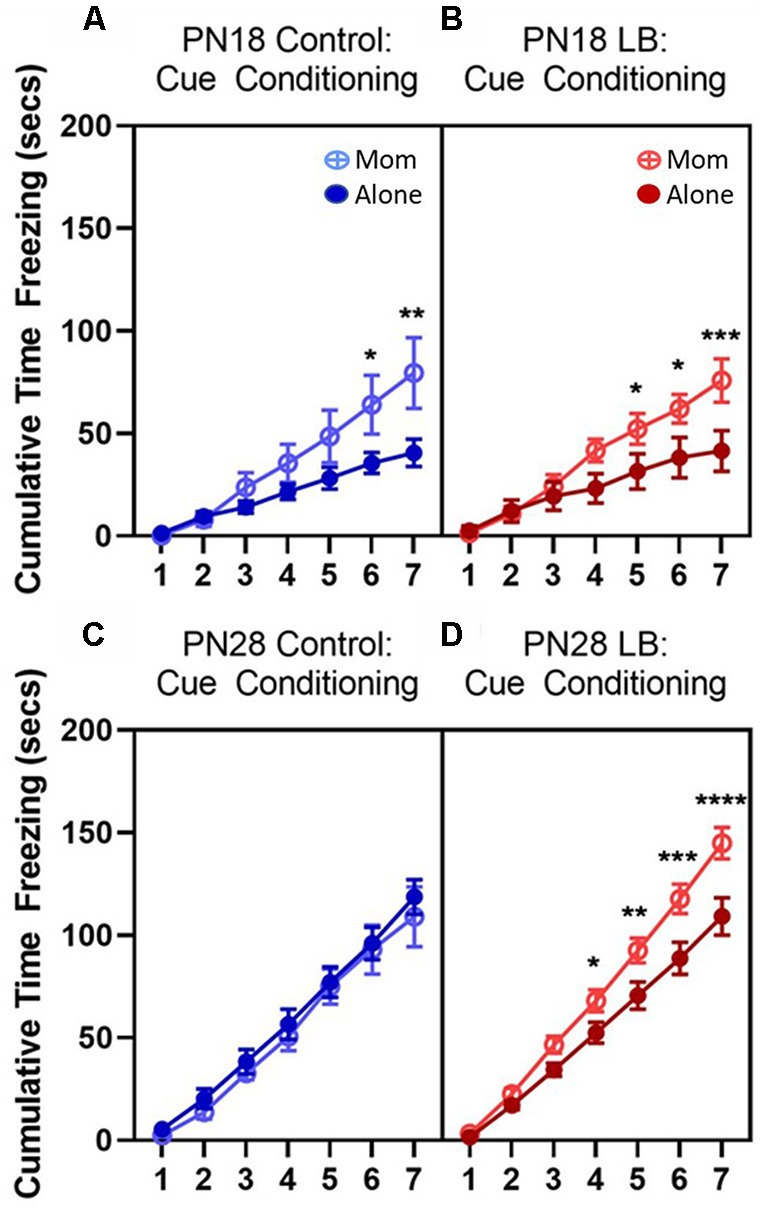
Odor-shock cue conditioning increases freezing to odor during training. Total cumulative (seconds) freezing (±SEM) during paired odor-shock cue conditioning at **(A,B)** PN18 (Control: Alone *n* = 8; Mom *n* = 8; LB: Alone *n* = 8; Mom *n* = 8) and **(C,D)** PN28 (Control: Alone *n* = 9; Mom *n* = 8; LB: Alone *n* = 14; Mom *n* = 15). Cue conditioning increased freezing at both ages in both control and low bedding (LB) rearing conditions however increased freezing was observed in animals conditioned with the mom (except in PN28 Control group) relative to animals conditioned alone. Open circle = conditioned with the mom; filled circles = conditioned alone; blue = controls; red = LB. *p < 0.05, **p < 0.01, ***p < 0.001, ****p < 0.0001.

Assessment of paired animals with and without maternal presence during conditioning revealed significantly higher freezing in animals conditioned with the mom relative to animals conditioned alone during later trials except in PN28 control animals (Figure 2). For PN18 controls (Figure 2A), there was no main effect of maternal presence (*F*_(1,14)_ = 2.601, *p* = 0.129) but there was a main effect of cue presentation (*F*_(6,84)_ = 37.90, *p* < 0.001) and a cue presentation by maternal presence interaction (*F*_(6,84)_ = 4.567, *p* = 0.0005). *Post hoc* tests showed that during the sixth (*p* = 0.016) and seventh (*p* = 0.001) odor presentations animals conditioned with the mother showed higher freezing relative to animals conditioned alone. For the PN18 LB group (Figure 2B), there also was no main effect of maternal presence (*F*_(1,14)_ = 2.885, *p* = 0.112) but there was a main effect of cue presentation (*F*_(6,84)_ = 50.66, *p* < 0.001) and a cue presentation by maternal presence interaction (*F*_(6,84)_ = 5.563, *p* < 0.001). Similar to the control animals, during later cue presentations [fifth (*p* = 0.040), sixth (*p* = 0.019) and seventh (*p* < 0.001)] animals conditioned with the mother showed higher freezing relative to animals conditioned alone.

At PN28, similar effects were observed in the LB group although there were fewer differences observed in the control animals (Figure 2C). For controls, there was no main effect of maternal presence (*F*_(1,15)_ = 0.331, *p =* 0.574) or cue presentation by maternal presence interaction (*F*_(6,90)_ = 0.177, *p* = 0.983), but there was a main effect of cue presentation (*F*_(6,90)_ = 167.0, *p* < 0.001). The increase in freezing over time did not differ between animals conditioned alone or with the mom. In contrast, in the PN28 LB group (Figure 2D) there was a main effect of maternal presence (*F*_(1,27)_ = 6.810, *p =* 0.015), cue presentation (*F*_(6,162)_ = 410.9, *p* < 0.001) and a cue presentation by maternal presence interaction (*F*_(6,162)_ = 7.722, *p* < 0.001). During the fourth (*p* = 0.046), fifth (*p* = 0.006), sixth (*p* < 0.001) and seventh (*p* = <0.001) odor presentations, animals conditioned with the mother showed higher freezing relative to animals conditioned alone.

### Cue Test

**Figure 3:**
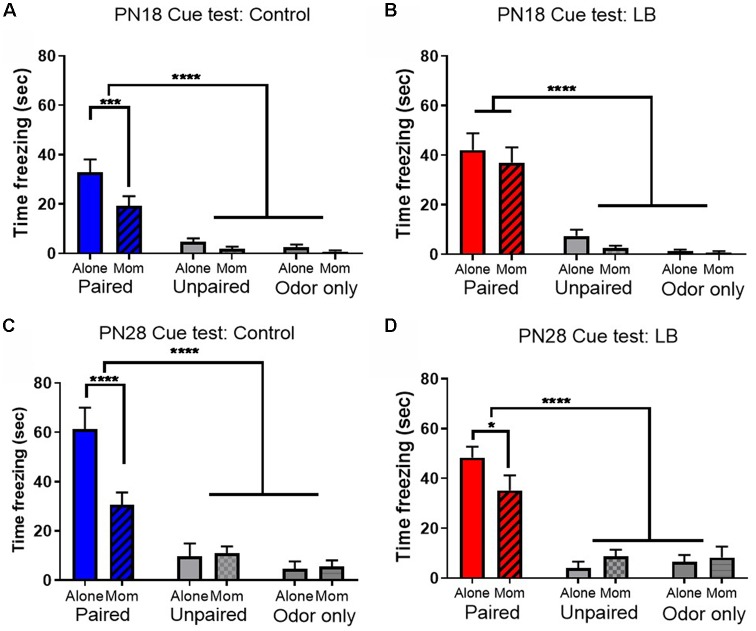
Early abuse modulates maternal buffering of odor-shock conditioning. Total (seconds) freezing (±SEM) to a conditioned stimulus (CS) was higher in Paired odor-shock conditions than Unpaired and Odor only conditions. Maternal presence during conditioning attenuated learning at both **(A,B)** PN18 Control (Paired: Alone *n* = 12; Mom *n* = 12; Unpaired: Alone *n* = 12; Mom *n* = 12; odor Only: Alone *n* = 12; Mom *n* = 12) and LB (Paired: Alone *n* = 11; Mom *n* = 12; Unpaired: Alone *n* = 12; Mom *n* = 12; odor Only: Alone *n* = 12; Mom *n* = 12) and **(C,D)** PN28 Control (Paired: Alone *n* = 9; Mom *n* = 10; Unpaired: Alone *n* = 8; Mom *n* = 8; odor Only: Alone *n* = 8; Mom *n* = 8) and LB (Paired: Alone *n* = 14; Mom *n* = 14; Unpaired: Alone *n* = 8; Mom *n* = 8; odor Only: Alone *n* = 8; Mom *n* = 8), although this maternal presence effect was not present following early life PN18 LB maltreatment and present but attenuated following early life PN28 LB maltreatment. *p < 0.05, ****p < 0.0001.

Overall, all paired animals at both ages and in both rearing conditions showed increased freezing to the CS relative to controls, indicating retention of the learned association between the odor and the shock (Figure 3; Johansen et al., 2011). For PN18 Controls (Figure 3A), there was a main effect of learning condition (*F*_(2,66)_ = 49.07, *p* < 0.001), maternal presence (*F*_(1,66)_ = 7.27 *p* = 0.009) and a trending interaction (*F*_(2,66)_ = 2.784, *p* = 0.069). *Post hoc* tests revealed that freezing in Paired groups with and without mom was significantly higher than control groups (all *p*’s < 0.05) and maternal presence increased paired group freezing relative to paired animals conditioned alone (*p* < 0.001). For the PN18 LB group (Figure 3B), there was a main effect of learning condition (*F*_(2,65)_ = 60.00, *p* < 0.001), no effect of maternal presence (*F*_(1,65)_ = 1.27, *p* = 0.264) nor an interaction effect (*F*_(2,65)_ = 0.22, *p* = 0.80). *Post hoc* tests revealed that paired group freezing with and without mom was significantly higher than control groups (all *p*’s < 0.05) and no significant difference between the two paired groups with and without the mother (*p* = 0.359) suggesting the mother did not suppress learning.

At PN28, similar effects were found for both controls and LB: only paired animals learned, although both rearing conditions showed attenuated learning with maternal presence. Specifically, for controls (Figure 3C) there were significant main effects of learning (*F*_(2,45)_ = 36.93, *p* < 0.001), maternal presence (*F*_(1,45)_ = 4.872, *p* = 0.032) and an interaction (*F*_(2,45)_ = 6.363, *p* = 0.004). *Post hoc* tests revealed that both paired freezing with and without the mom was significantly higher than all control groups (*p*’s < 0.05) and there was a significant difference between the two paired groups (with mother freezing increased relative to without the mother, *p* < 0.01). A similar behavioral pattern was found in LB-reared animals (Figure 3D); there was a main effect of learning condition (*F*_(2,54)_ = 39.81, *p* < 0.001), no effect for maternal presence (*F*_(1,54)_ = 0.311, *p* = 0.579) nor was there an interaction (*F*_(2,54)_ = 2.215, *p* = 0.119). *Post hoc* tests revealed that the paired groups with and without mom were significantly higher than all control groups (*p*’s < 0.001) and maternal presence increased paired group freezing (with mother vs. without the mother, *p* = 0.026).

### Neural Analysis of Prefrontal Cortex (PFC)

Overall, we found significant evidence that PFC activation in several subregions at PN28 varied as a function of rearing condition (Control and LB) and whether the mother was present during conditioning. In contrast, no such PFC activation patterns at age PN18 were observed.

#### Infralimbic Prefrontal Cortex (IL)

**Figure 4:**
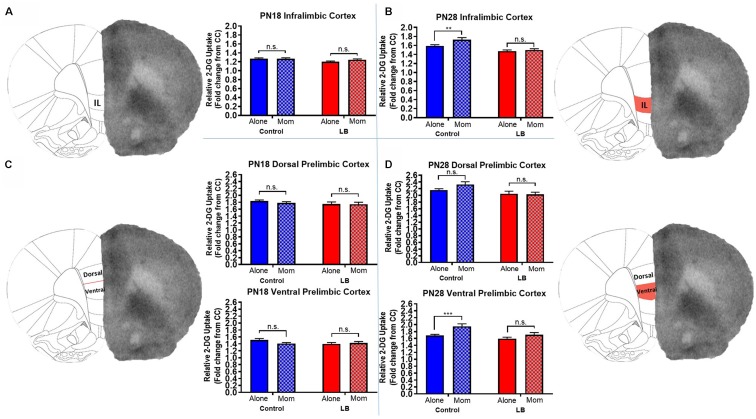
Previous abuse impairs maternal regulation of vmPFC regions during threat in older pups. Average (±SEM) fold change in activity for **(A,C)** PN18 Control infralimbic (IL: Alone *n* = 40; Mom *n* = 44; dorsal PL: Alone *n* = 41; Mom *n* = 33; ventral PL: Alone *n* = 30; Mom *n* = 33) and LB (IL: Alone *n* = 28; Mom *n* = 28; dorsal PL: Alone *n* = 28; Mom *n* = 21; ventral PL: Alone *n* = 21; Mom *n* = 21) or **(B,D)** PN28 Control (IL: Alone *n* = 32; Mom *n* = 28; dorsal PL: Alone *n* = 24; Mom *n* = 24; ventral PL: Alone *n* = 32; Mom *n* = 32) and LB (IL: Alone *n* = 32; Mom *n* = 298; dorsal PL: Alone *n* = 24; Mom *n* = 27; ventral PL: Alone *n* = 32; Mom *n* = 36). Solid bars represent pups conditioned alone while hashed bars represent pups conditioned with the mother. Right, schematics of brain regions; red, subregions with statistically significant effect of abuse on 2-DG uptake. n.s., indicates a non-significant effect (*p* > 0.05); **p < 0.01; ***p < 0.001.

The PFC showed significant differences across the dorsal-ventral axis at PN28, but not at PN18. Specifically for PN28 animals (Figure 4B), IL 2-DG uptake was higher when pups received paired CS-US conditioning with the mother vs. conditioned alone, while abused pups failed to show this effect [two-way ANOVA (rearing × maternal presence): main effect of rearing (*F*_(1,117)_ = 30.77, *p* < 0.0001), main effect of maternal presence (*F*_(1,117)_ = 6.754, *p* = 0.011), and a trending interaction (*F*_(1,117)_ = 2.965, *p* = 0.088)]. *Post hoc* tests showed that maternal presence during paired odor-shock conditioning was associated with increased 2-DG uptake in IL in controls, but not LB-reared PN28 pups (control alone vs. control with mom, *t*_(117)_ = 3.041, *p* = 0.003; LB alone vs. LB with mom, *t*_(117)_ = 0.623, *p* = 0.535).

At PN18 (Figure 4A), we failed to observe an effect of maternal presence on 2-DG uptake, though a main effect of rearing was observed [two-way ANOVA (rearing × maternal presence), main effect of rearing (*F*_(1,136)_ = 4.705, *p* = 0.032); no main effect of maternal presence (*F*_(1,136)_ = 1.127, *p* = 0.293); no interaction (*F*_(1,136)_ = 1.557, *p* = 0.214)]. *Post hoc* tests showed that LS-reared pups exhibited lower 2-DG uptake levels compared to controls (LS with mom vs. control with mom, *t*_(136)_ = 2.44, *p* = 0.016; LB with mom vs. control alone, *t*_(136)_ = 2.26, *p* = 0.025).

#### Prelimbic Prefrontal Cortex (PL)

We observed that in PN28 pups (Figure 4D), the ventral region of the PL showed significant changes in 2-DG metabolism depending on rearing condition (Control vs. LS) as well as maternal presence [two-way ANOVA (rearing × maternal presence), main effect of rearing (*F*_(1,128)_ = 9.127, *p* = 0.003), main effect of maternal presence (*F*_(1,128)_ = 12.13, *p* = 0.001), no interaction (*F*_(1,128)_ = 1.902, *p* = 0.170)]. *Post hoc* tests showed that maternal presence increased activity in Control pups conditioned with the mom but not LB (control alone vs. control with mom, *t*_(128)_ = 3.39, *p* = 0.001; LB alone vs. LB with mom (*t*_(128)_ = 1.51, *p* = 0.134). In the dorsal PL, only a main effect of rearing was observed (*F*_(1,95)_ = 9.045, *p* = 0.003) with no main effect of maternal presence (*F*_(1,95)_ = 1.288, *p* = 0.259) or interaction (*F*_(1,95)_ = 1.869, *p* = 0.175). 2-DG uptake was decreased in all LS-reared groups compared to controls (LS with mom vs. control with mom, *t*_(95)_ = 3.138, *p* = 0.002; LB alone vs. control mom, *t*_(95)_ = 2.888, *p* = 0.005). At PN18 (Figure 4C), dorsal PL had no effects of maternal presence, rearing, or an interaction observed in the ventral (*F*_(1,101)_ = 1.066, *p =* 0.304, *F*_(1,101)_ = 1.764, *p =* 0.1871, and *F*_(1,101)_ = 3.199, *p* = 0.076, respectively) or dorsal (*F*_(1,119)_ = 0.480, *p* = 0.490, *F*_(1,119)_ = 1.831, *p* = 0.1785, *F*_(1,119)_ = 0.243, *p =* 0.623, respectively).

## Discussion

**Figure 5:**
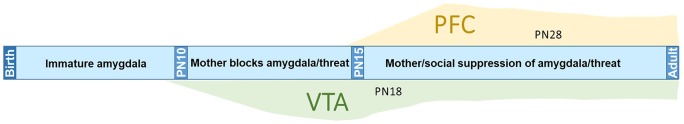
Developmental transitions in amygdala inputs regulating social suppression of threat. The neural circuit supporting infant fear learning and its maternal presence blockade (≤PN15) or attenuation (≥PN16) undergoes developmental changes. Social buffering in early infancy is supported by VTA-amygdala connectivity (≤PN15), while in older pups (PN28) and adults social attenuation of fear is supported by vmPFC-amygdala connectivity. This system is disrupted following early life abusive rearing. At PN18, the ability of the mother to block fear learning is abolished and VTA showed compromised suppression of the amygdala. Early life maltreatment leaves social suppression of fear learning intact at PN28, although it is effectiveness is reduced and social modulation of vmPFC engagement is significantly reduced.

Here, we assessed the neurobiology of social buffering of threat learning in typical and perturbed development. We focused on a developmental transition from dependence on the mother (PN18) to independence in preadolescent rats (PN28) weaned from the mother. Overall, our results show that social buffering of threat occurs across the lifespan, although the underlying neural circuit diverges, with the present results suggesting a late emerging role for the PFC after weaning from the mother. We summarize these results and integrate them into the existing social buffering literature in Figure 5: maternal presence blocks fear learning in early development, but switches to attenuation of threat responding, which behaviorally appears similar from PN16 into adulthood. This system is disrupted by early life trauma: PN18 maltreated pups were not socially buffered by the mother, but social buffering of threat emerged again by PN28. Most surprisingly, expression of social buffering in maltreated preadolescents did not require PFC engagement. Taken together, these results suggest that social buffering is a dynamic process that is sensitive to developmental events in an age-dependent manner.

Using an age range when the PFC and its connectivity with the amygdala are maturing (Bouwmeester et al., 2002; Cressman et al., 2010; Willing and Juraska, 2015; Arruda-Carvalho et al., 2017), we asked if the PFC is involved in maternal suppression of fear learning in infant rats during a developmentally significant transitional period. In humans, the late-developing PFC shows a switch from positive to negative connectivity with the amygdala as children develop into adolescents and amygdala-prefrontal circuitry is associated with increased behavioral modulation of children by their mothers (Gee et al., 2013b, 2014). Furthermore, early life trauma is associated with dysregulated cortico-limbic network connectivity through adolescence, impaired stress responding, and cortico-limbic hyperactivity in response to negative social cues (Andersen and Teicher, 2008; Suzuki et al., 2014; Teicher et al., 2014, 2016; Kaiser et al., 2018). Together with the current results, these reports suggest that prefrontal modulation of interacting fear and social systems contributes to the developmental profile of maternal fear regulation and this system can be disrupted following early life trauma. However, further investigation is needed to confirm this hypothesis.

### Typical Rearing: Social Buffering of Threat Occurs at Both PN18 and PN28, but the PFC Is Only Engaged in Newly Independent PN28 Pups

The similar social buffering effects on the behavioral level at PN18 and PN28 appear to be supported by different neural networks; the ventromedial (vm)PFC IL and PL subregions were only modulated by the mother in the PN28 animals. The PFC is a late-developing structure (Gee et al., 2013b; Schubert et al., 2015; Hennessy et al., 2018) and the older infant/child and adult literature validates the important role of the amygdala and vmPFC for social buffering in humans (Lungwitz et al., 2014; Hornstein et al., 2016; Hornstein and Eisenberger, 2017; van Rooij et al., 2017), nonhuman primates (Winslow et al., 2003; Suomi et al., 2008; Sanchez et al., 2015; Howell et al., 2017) and rodents (Hennessy et al., 2015; Penha Farias et al., 2019). The absence of a PFC effect in the youngest pups is consistent with the literature as well. These reports suggest that the rodent vmPFC is not engaged by simple maternal presence, simple innate threat presentation, or learning about threat until around weaning age (~PN23; Kim et al., 2009; Chan et al., 2011; Ball and Slane, 2012; Li et al., 2012; Shechner et al., 2014; Takahashi, 2014; Almada et al., 2015; Perry et al., 2016; Heroux et al., 2017; Robinson-Drummer et al., 2018). It should be noted that the PFC appears to be involved in the appetitive system in PN18 pups (Lilliquist et al., 1999; Nair et al., 2001a,b), suggesting a staggered developmental functional onset for various PFC functions.

The newly emerging role of the vmPFC by PN28 to support social buffering of threat is consistent with vmPFC importance in adult fear conditioning social presence literature in humans and rodents. For example, in adult rats, the presence of a cage mate significantly attenuates fear learning, compared to those conditioned alone and engages the vmPFC (Kiyokawa et al., 2014, 2007; Penha Farias et al., 2019). This effect also occurs in humans and involves the vmPFC; in adults, the presence of an important social partner (i.e., mother, romantic partner, cage mate) or a stimulus that provokes the memory of an individual (i.e., odor, photo) dampens fear through amygdala-vmPFC to block adult fear learning across species (Guzmán et al., 2009; Fuzzo et al., 2015; Hornstein et al., 2016; Hornstein and Eisenberger, 2017; van Rooij et al., 2017; Toumbelekis et al., 2018). Our results also overlap with the literature involving non-social cues predicting safety within a threatening situation: conditioned inhibitors/safety signals use a similar network of PFC input suppressing the amygdala (Rogan et al., 2005; Pollak et al., 2008; Christianson et al., 2012; Harrison et al., 2017; Levin et al., 2017). The specific connection between the vmPFC and amygdala has not been documented within the social buffering of threat literature, although our general understanding of vmPFC-amygdala functional connectivity suggests the PFC is required to modulate the amygdala’s output response to threat (Phelps et al., 2004; Corcoran and Quirk, 2007; Marek et al., 2013). In general, the IL appears to reduce fear (Quirk et al., 2000; Sotres-Bayon et al., 2004; Do-Monte et al., 2015), and this is consistent with our findings; the largest maternal response in the PFC was found in the IL. In contrast, the PL is generally associated with enhanced amygdala responding and enhanced amygdala-dependent response to threat (Sharpe and Killcross, 2015a,b, 2018; Ye et al., 2017) although prelimbic-infralimbic projections have been shown to contribute to reductions in fear expression (Marek et al., 2018). The dorso-ventral gradient of activity observed at PN28 support a role for a subset of PL contributing to fear reduction with ventral regions sharing function with the IL cortex; a finding not surprising due to their close anatomical proximity.

As we consider the functional significance of late PFC engagement by social buffering of threat during early life, we suggest that as pups leave the nest they encounter a far more complicated environment where higher order brain areas (such as the vmPFC) are required for processing complex threat and safety cues. Indeed, outside the nest an animal must use changing, context- and time-dependent safety/threat cues to choose appropriate approach/avoidance responses in environments with complex social hierarchy (Cunningham et al., 2002; Holland and Gallagher, 2004; Taylor et al., 2008; Maren et al., 2013; Opendak et al., 2017). Development of functional connectivity between the vmPFC, threat and social circuits would allow necessary integration of these cues thereby facilitating proper social interactions and threat evaluation.

### Maltreatment Rearing Blocked Social Buffering of Threat at PN18, but Returns at PN28 Without PFC Engagement

One of the more intriguing aspects of the present data is the effect of rearing on social buffering at across development; early life maltreatment transiently suppressed social buffering at PN18 (replicating effects observed in Opendak et al., 2019) and social buffering returned at PN28. Our experiments do not suggest a mechanism for this transition, although the evidence points to the slow decline of the infant VTA social buffering system and the protracted emergence of the adult-like, PFC-dependent, social buffering system (see Figure 5). Specifically, our previous work suggests this PN18 maltreatment effect is due to disruption of the infant VTA dopaminergic input to the basolateral amygdala, the mechanism supporting social blockade and suppression of fear learning in younger pups (Barr et al., 2009; Opendak et al., 2019). In further support of this framework, in typically-reared PN28 pups, social buffering was associated with PFC engagement, which was not observed at PN18.

Another striking feature of these data is the dissociation between PFC and social buffering following maltreatment in preadolescents. Specifically, buffering was still observed at PN28 following maltreatment, though we failed to observe the engagement of the PFC documented in control-reared pups. We should note that early maltreatment seemed to reduce the effect of maternal presence on fear learning; LB pups showed a smaller difference in freezing between paired conditioning alone and with mom groups although this result requires replication and direct comparison in a future study. However this complements existing literature on the impact of early life stress on the infant PFC and infant learning (Callaghan and Richardson, 2012; Pattwell et al., 2012; Fareri et al., 2017; Peña et al., 2017; Bath, 2018; Callaghan et al., 2019; Junod et al., provisionally accepted) and extends these results to include reduced social reduction of fear.

While it is abundantly clear that early life stress disrupts pups’ neurobehavioral development (Barbosa Neto et al., 2012; Tang et al., 2014; Doherty and Roth, 2016; Pattwell and Bath, 2017; Walker et al., 2017), including PFC development (Braun and Bock, 2011; Kunzler et al., 2015; Schubert et al., 2015; Hanson et al., 2018; VanTieghem and Tottenham, 2018), we speculate that a critical feature of this effect is that the ability of the mother to impact pups’ brains has failed to acquire the strength or value it has in typically reared pups. Indeed, our previous assessment of the value of maternal odor in control-reared vs. maltreatment-reared pups shows a slight yet significant decrease in approach to the maternal odor and decreased activation of amygdala and PFC in response to a maternal odor presentation without threat (Perry et al., 2016). It should be noted that the maltreatment-associated maternal odor *increases* in value across development. Indeed, adults reared with maltreatment have greater reduction of threat by maternal odor compared to controls, as evidenced by suppression of amygdala, attenuated fear conditioning and normalization of depressive-like behaviors (Sevelinges et al., 2011; Rincón-Cortés et al., 2015). This phenomenon may contribute to the transient effect of LB on behavior between PN18 and 28; as weaned animals approach adulthood, the weakened maternal cue naturally regains value and is able to reduce fear behavior. This would suggest that modulation of LB fear behavior is redirected through other circuit nodes (e.g., the VTA) when maternal presence fails to modulate vmPFC activity at this age. Thus, in addition to the social buffering network changing during development, the social signal processing within a larger social brain network may contribute to maltreatment-associated effects on social buffering.

## Conclusion

As we consider the implications of these results for infant neurobehavioral development and integration with the broader human development work, this work may inform our understanding of attachment. Within Attachment Theory, the mother is considered a “safe haven” or a source of safety, wherein the infant approaches the caregiver for safety and the caregiver reduces fear (Kerns et al., 2015; Hornstein et al., 2016). Here, using a fear conditioning paradigm, we show that maltreatment diminishes the mother’s ability to serve as a “safe haven” and social buffering of threat takes on a nonlinear effect across development.

## Data Availability

All datasets generated for this study are included in the manuscript.

## Ethics Statement

This study was carried out in accordance with the recommendations of National Institutes of Health. The protocol was approved by the Institutional Animal Care and Use Committees.

## Author Contributions

RS, MO and PR-D designed the experiments. PR-D, KW, MO and RS conducted the research/analyzed behavior. MO and AB made illustrations. ST, AB, PR-D, MO, SC, EF, CS, AS, DC, CD and ST analyzed the IA data. KW, LJ and GK performed histology and autoradiography. RS, PR-D, MO and AB wrote the manuscript. PR-D, MO, AB and RS performed and were consulted on data analysis and statistics.

## Conflict of Interest Statement

The authors declare that the research was conducted in the absence of any commercial or financial relationships that could be construed as a potential conflict of interest.
